# Efficacy of erector spinae plane block for postoperative analgesia lumbar surgery: a systematic review and meta-analysis

**DOI:** 10.1186/s12871-023-02013-3

**Published:** 2023-02-16

**Authors:** Qianchuang Sun, Chengwei Zhang, Shuyan Liu, Hui Lv, Wei Liu, Zhenxiang Pan, Zhimin Song

**Affiliations:** 1 Department of Anesthesiology, The Second Hospital of Jilin University, Changchun, 130041 China; 2 Department of Ophthalmology, The Second Hospital of Jilin University, Changchun, 130041 China

**Keywords:** Lumbar surgery, Meta-analysis, Opioid consumption, Pain scores, Erector spinae plane block

## Abstract

**Objectives:**

The erector spinae plane (ESP) block is a newly defined regional anesthesia technique first described in 2016. The aim of this meta-analysis is to assess the efficacy of ESP block in improving analgesia following lumbar surgery.

**Methods:**

PubMed, EMBASE, Cochrane Library, and Web of Science were searched for randomized controlled trials (RCTs) that compared the analgesic efficacy of the ESP block with non-block care for lumbar surgery from inception 3 August 2021. The primary outcomes were postoperative opioid consumption and pain scores during the first 24 h. Postoperative pain was measured as pain at rest and on movement at postoperative 0, 4, 8, 12, and 24 h expressed on a visual analog scale (VAS), where 0 = no pain and 10 = the most severe pain.

**Results:**

11 studies involving 775 patients were included in our analysis. The use of ESP block significantly decreased 24-h opioid consumption (WMD, -8.70; 95% CI, -10.48 to -6.93; I^2^ = 97.5%; *P* < 0.001) compared with the non-block. Moreover, ESP block reduced pain scores at postoperative time-points up to 24 h. ESP block also prolonged the time to first analgesic request (WMD = 6.93; 95% CI: 3.44 to 10.43, I^2^ = 99.8%; *P* < 0.001). There was less PONV with ESP block versus non-block group (RR, 0.354; 95% CI, 0.23 to 0.56; I^2^ = 25.2%; *P* < 0.001), but no difference in pruritus.

**Conclusions:**

ESP block provides less opioid consumption and PONV, lower pain scores, and longer time to first analgesic request in patients undergoing lumbar surgery compared to general anesthesia alone.

**Supplementary Information:**

The online version contains supplementary material available at 10.1186/s12871-023-02013-3.

## Introduction

Posterior lumbar surgery is associated with severe postoperative pain, which typically persists for the initial few days [1]. Such patients usually require significant amounts of intravenous opioids during the first few days, which increase opioid-related complications [2]. Severe postoperative pain reduces patients' satisfaction with surgery and can delay postoperative recovery, ambulation, and discharge from the hospital [3]. Thus, efficient and safe methods for postoperative analgesia after lumbar spine surgery are beneficial for early recovery.

Regional anesthesia, as part of a multimodal approach, would seem to be one of ideal choices for addressing spine surgery pain management, such as paravertebral block, interfacial plane block, neuraxial technique, and local anesthetic wound infiltration [4]. While the widespread adoption of these techniques is hampered due to several drawbacks. The benefit of local anesthetic wound infiltration tends to be short-lived [5]. The erector spinae plane (ESP) block is a newly defined regional anesthesia technique first described in 2016 [6]. Forero et al. proposed that the administration of local anesthetic into the plane between the deep fascia of the erector spinae muscle and the vertebral transverse process produced an extensive sensory block over the ipsilateral thorax [6]. Thanks to safe and easy to perform under ultrasound guidance, the ESP block has been used as postoperative analgesia in different types of surgery, such as abdominal, thoracic, breast, and spinal surgeries [6–9]. Despite the evidence, there is still a debate regarding the mechanism of action and efficacy of this new technique. Previous meta-analyses investigating the analgesic efficacy of ESP block for breast and thoracic surgery patients have shown that ESP block is more effective at reducing postoperative opioid consumption and pain scores compared with non-block care [10–13]. In addition, a recent meta-analysis indicated that ESP block significantly reduced opioid consumption and relieved postoperative pain after lumbar spinal surgery [14]. However, the analysis was underpowered since only six studies were included [14]. Therefore, we performed this meta-analysis to reappraise the literature in order to determine the analgesic efficacy of ESP block for lumbar surgery in adult patients. We included randomized controlled trials (RCTs) comparing the ESP block with general anesthesia (GA) alone. 24-h postoperative opioid consumption and postoperative pain scores were defined as primary outcomes.

## Materials and methods

We performed this study according to the Preferred Reporting Items for Systematic Reviews and Meta-Analyses (PRISMA) recommendations (Supplementary Table 1) [15]. The study was not registered with the International Prospective Register of Systematic Reviews (PROSPERO).

### Literature search

We systematically searched the PubMed, EMBASE, Cochrane Library, and Web of Science databases from inception to 3 August 2021. The search terms were “bilateral erector spinae plane block” and “lumbar surgery”. The details of the search strategies are summarized in Supplementary Table 2. The search was restricted to articles in the English language. In order to identify any other potentially relevant trials, we manually searched for conference summaries and references for potential eligible reports.

### Types of comparisons

Comparisons will be made between the experimental (ESP block) group and the control (non-block care) group.

### Types of outcomes

The primary outcomes were 24-h postoperative opioid consumption and postoperative pain scores. The secondary outcomes were first request for analgesia, side effects, and block-related complications.

### Types of study designs

Inclusion criteria were as follows: (1) studies designed as RCTs; (2) patients undergoing open posterior lumbar surgery, including lumbar decompression, lumbar spinal fusion, lumbar discectomy, lumbar laminectomy, and lumbar fixation; (3) experimental groups treated with GA plus bilateral ESP block, and the control group with GA alone; (4) outcomes such as pain scores, postoperative opioid consumption, intra-operative opioid consumption, time to first request for analgesia, and opioid-related side effects.

Exclusion criteria were as follows: (1) non-RCTs; (2) letters, reviews, comments, editorials, abstracts, technical reports and case reports; (3) no control group.

### Data extraction

Two authors (QCS, CWZ) independently collected data from all included studies. Disagreements were resolved by consultation with a third author (ZMS). Extracted data included author; year; sample size; local anesthetic used; block technique; postoperative analgesic use; postoperative pain scores at rest and on movement; time to first analgesic request; and incidence of side effects. We also recorded complications related to the erector spinae plane block. 24-h postoperative opioid consumption and postoperative pain scores were defined as primary outcomes. Secondary outcomes were first request for analgesia, side effects, and block-related complications. If data were presented as median and interquartile range (IQR), we contacted the author for necessary data. Failing that, we used formulas to estimate the mean and standard deviation [16]. If nausea and vomiting were reported as separate measures, we used nausea data to avoid double counting. To standardize outcome metrics, all reported perioperative opioid consumption was converted to postoperative morphine equivalent doses [17, 18].

### Assessment of quality and bias

The Cochrane Risk of Bias Tool was performed to assess the quality of the included studies [19]. The evaluation should include the following domains: (1) random sequence generation; (2) allocation concealment; (3) blinding of participants and personnel; (4) blinding of outcome assessment; (5) incomplete outcome data; (6) selective reporting; (7) other bias. Each of these domains was judged as low risk, high risk, or unclear risk. Any disagreements were resolved by discussion.

For the assessment of publication bias of the studies included in the final analysis, both Begg’s rank correlation and Egger’s linear regression tests were performed [20, 21].

### Data analysis

All statistical analyses were performed in Stata software, version 15.0 (Stata Corp, College Station, Texas) and Review Manager, version 5.4 (The Nordic Cochrane Centre, The Cochrane Collaboration, Copenhagen, Denmark). Risk ratios (RRs) with 95% confidence intervals (CIs) were calculated for dichotomous data, and weighted mean differences (WMDs) with 95% CIs were calculated for continuous variables. Heterogeneity was assessed statistically using the χ2 standard test. Heterogeneity between studies was estimated using the Cochrane Q test and I^2^ index. Due to the included studies are few, the random-effects model with Hartung Knapp adjustments was applied in this meta-analysis [22, 23]. Subgroup analysis was performed for the outcome measures, according to time of block (before induction or after induction). Sensitivity analysis was performed by excluding one study each time to evaluate the influence of a single study on the overall estimate [24].

## Results

### Literature search

**Fig. 1:**
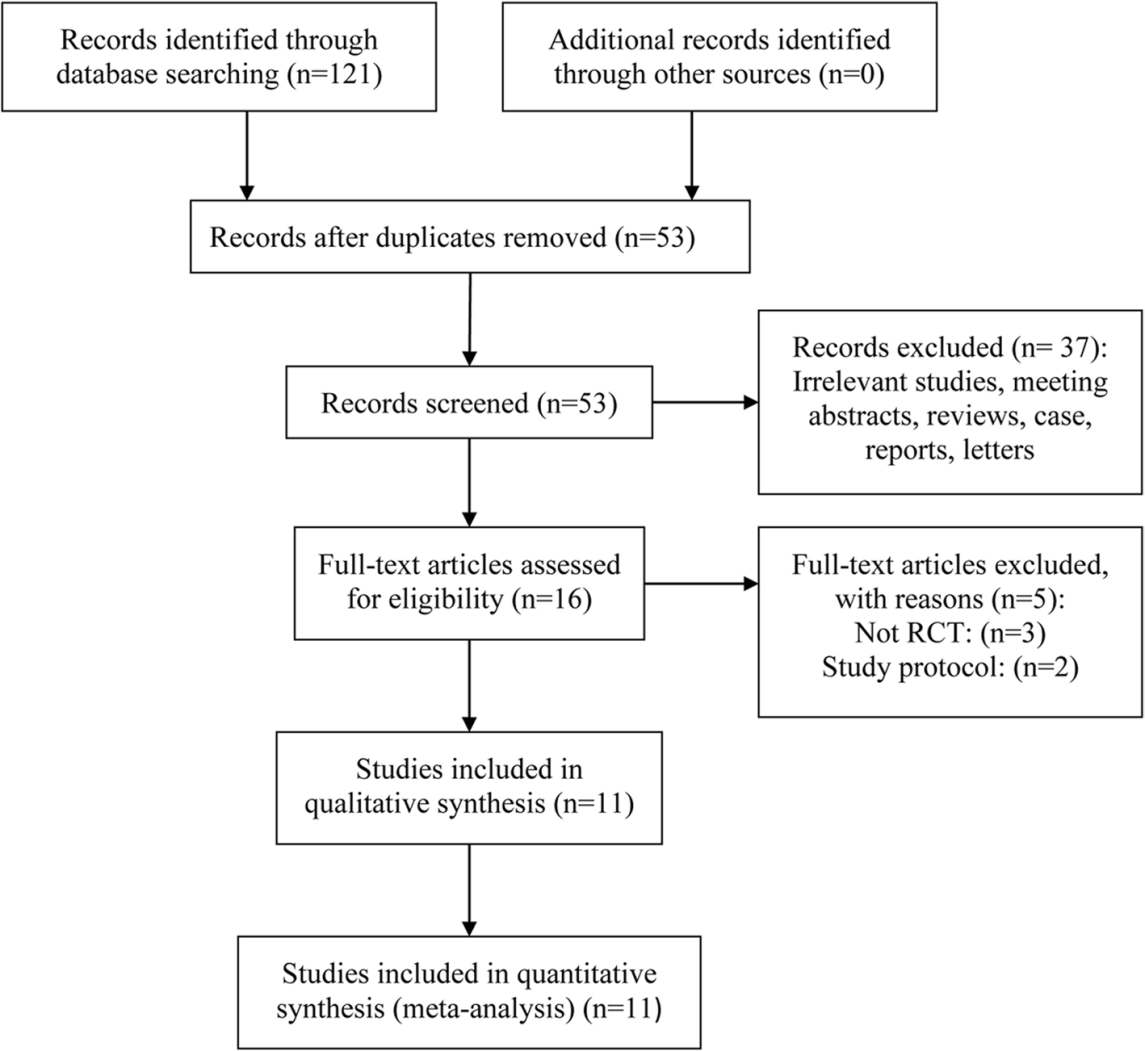
Flowchart of the study selection

A total of 121 studies were identified through database searching. Of these, 105 were excluded after excluding duplicate references and reviewing titles and abstracts. A further five trials were discarded upon study protocol (*n* = 2) or not RCT (*n* = 3). The reasons for excluded studies were presented in Supplementary Table 3. The remaining 11 studies that consisted of 775 patients were included in the systematic review [25–35]. The search process is provided in Fig. 1.

### Trial characteristics and study quality

**Fig. 2:**
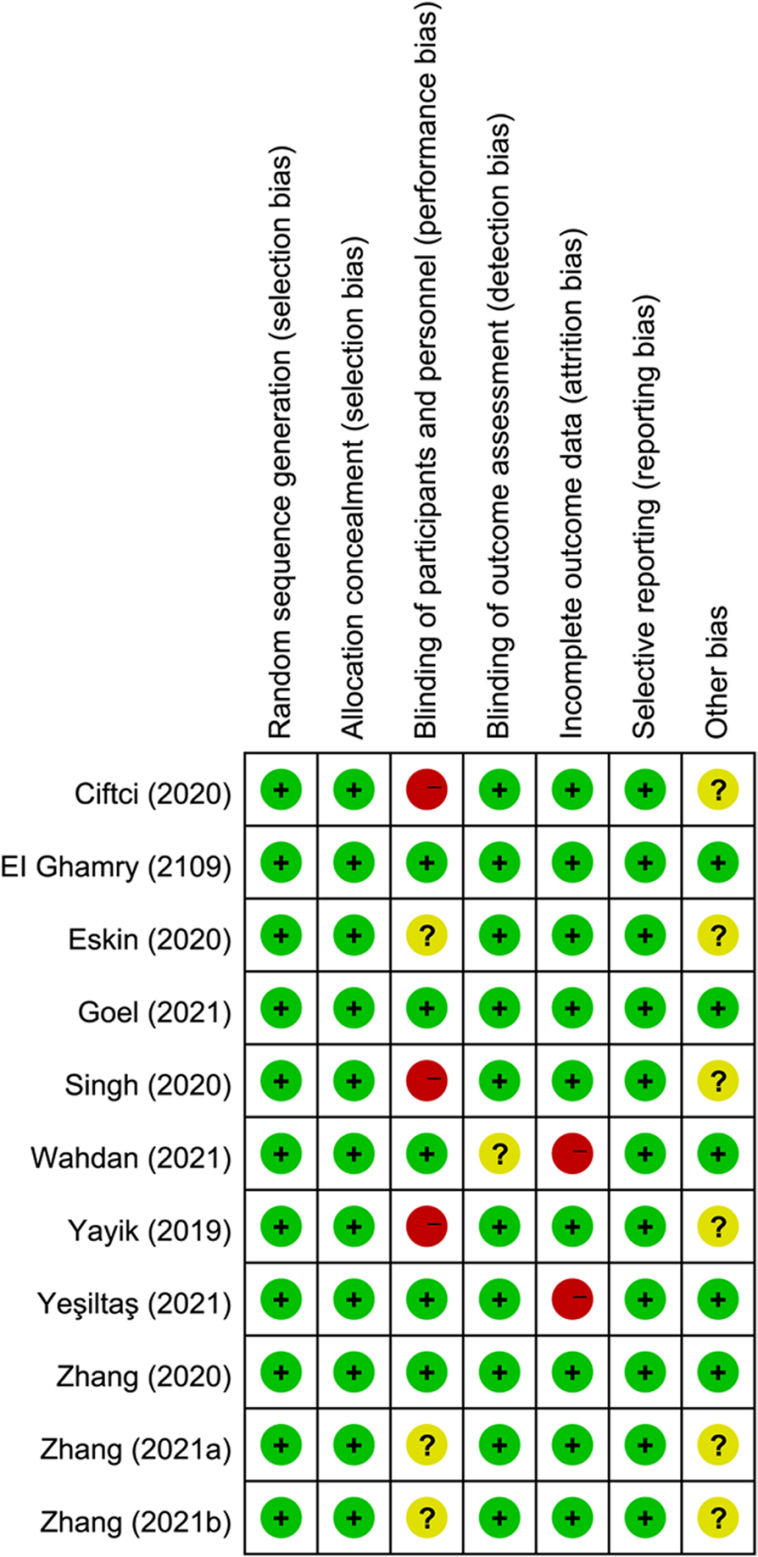
Risk of bias summary

The characteristics of the included studies are shown in Table 1. GA was performed in all studies. Of these 11 trials, six performed before the induction of GA [25–27, 30, 31, 34], while five performed ESP block after the induction of anesthesia [28, 29, 32, 33, 35]. Bupivacaine was the local anesthetic of choice in six trials [26, 28, 31, 33–35], ropivacaine in three studies [25, 27, 30], bupivacaine and lidocaine mixture in one study [32], and levobupivacaine in one study [29]. For postoperative analgesia, patient-controlled intravenous analgesia (PCIA) with morphine was provided in four studies [25, 29, 30, 32], PCIA with tramadol used in two studies [31, 33], PCIA with sufentanil provided in one study [27], and PCIA with fentanyl used in one study [35]. Pain scores were reported in all included trials. Six studies reported pain scores at rest and on movement,[25, 27, 30, 31, 34, 35] while the other five reported pain scores at rest [26, 28, 29, 32, 33]. However, it was not possible to use data from every trial due to inconsistencies in the way the data was presented or the pain symptoms described. Most trials had a low risk of bias, as well as several elements representing unclear or high risk of bias. The risk assessment of the included studies is presented in Fig. 2.

**Table 1:** Overview of included studies’ characteristics

**Author (year)**	Sample size		ESP block				CTRL block	Postoperative analgesia
	ESPB	CTRL	Timing	LA	Volume (ml)	Location		
Zhang (2020) [25]	30	30	Pre-induction	0.3% ropivacaine	25 + 25	T12	Ultrasound scan	Morphine PCIA
Singh (2020) [26]	20	20	Pre-induction	0.5% bupivacaine	20 + 20	T10	No block	Diclofenac, morphine
Zhang (2021a) [27]	30	30	Pre-induction	0.4% ropivacaine	20 + 20	L3	Subcutaneous infiltration (1 ml 1% lidocaine)	Sufentanil PCIA
Goel (2021) [28]	50	50	Post-induction	0.25% bupivacaine	20 + 20	Surgical level	No block	Paracetamol, Ketorolac, pregabalin capsule, fentanyl
Wahdan (2021) [29]	70	70	Post-induction	0.25% levobupivacaine	20 + 20	Operating level	20 ml of normal 0.9% saline	Ketorolac, morphine PCIA
Zhang (2021b) [30]	30	29	Pre-induction	0.3% ropivacaine	25 + 25	T10	No block	Morphine PCIA
Yayik (2019) [31]	30	30	Pre-induction	0.25% bupivacaine	20 + 20	L3	No block	Tramadol PCIA
Yeşiltaş (2021) [32]	28	28	Intraoperative freehand	0.25% bupivacaine and 1.0% lidocaine	20 + 20	Spinal instrumented levels	20 ml physiological saline	Paracetamol, morphine PCIA
Eskin (2020) [33]	40	40	Post-induction	0.25% bupivacaine	20 + 20	T12-L5	No block	Paracetamol, dexketoprofen, tramadol PCIA
EI Ghamry (2019) [34]	30	30	Pre-induction	0.25% bupivacaine	20 + 20	L3	No block	Paracetamol, ketorolac, morphine
Ciftci (2020) [35]	30	30	Post-induction	0.25% bupivacaine	20 + 20	L3	No block	Fentanyl PCIA, meperidine

## 24-h postoperative opioid consumption

**Fig. 3:**
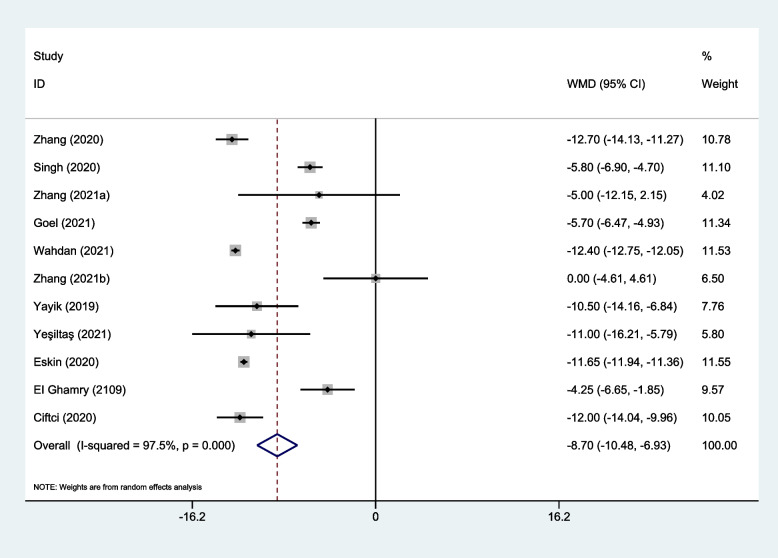
Forest plot of 24-h opioid consumption. CI = confidence interval, WMD = weighted mean difference

**Fig. 4:**
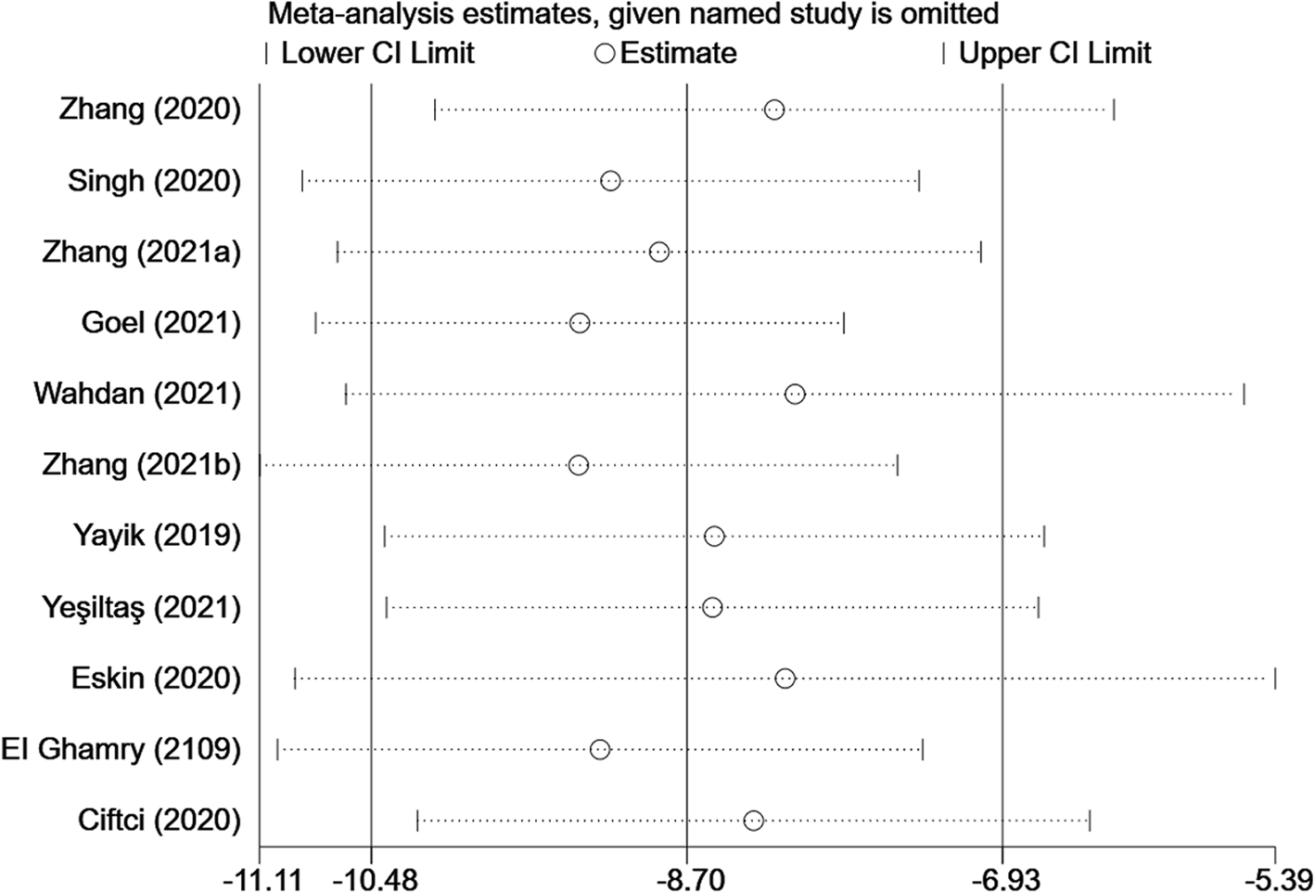
Sensitivity analysis of 24-h opioid consumption. CI = confidence interval

**Fig. 5:**
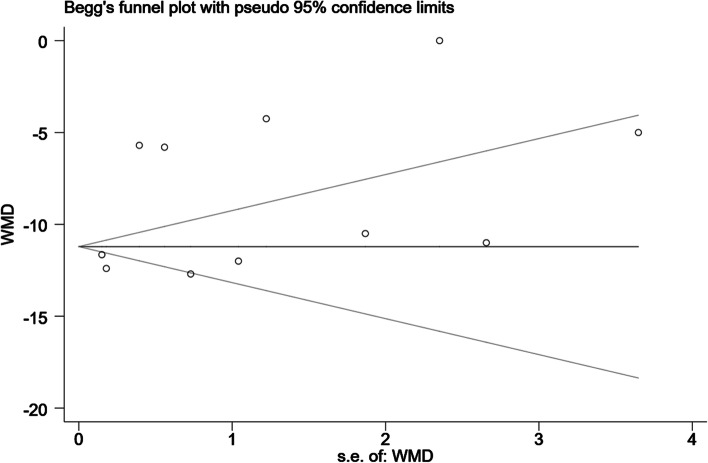
Begg’s funnel plot of 24-h opioid consumption. WMD, weighted mean difference

Eleven trials provided opioid consumption data in the first 24 h after surgery [25–35]. Meta-analysis revealed that ESP block might significantly reduce 24-h opioid consumption (WMD, -8.70; 95% CI, -10.48 to -6.93; I^2^ = 97.5%; *P* < 0.001) compared with the non-block groups (Fig. 3). Additional subgroup analysis of time of block (Fig. S1) as well as sensitivity analysis did not affect the pooled results (Fig. 4). The Begg’s funnel plot (*P* = 0.53) and the Egger’s test (*P* = 0.13) found no evidence of publication bias (Fig. 5).

## Postoperative pain scores

**Fig. 6:**
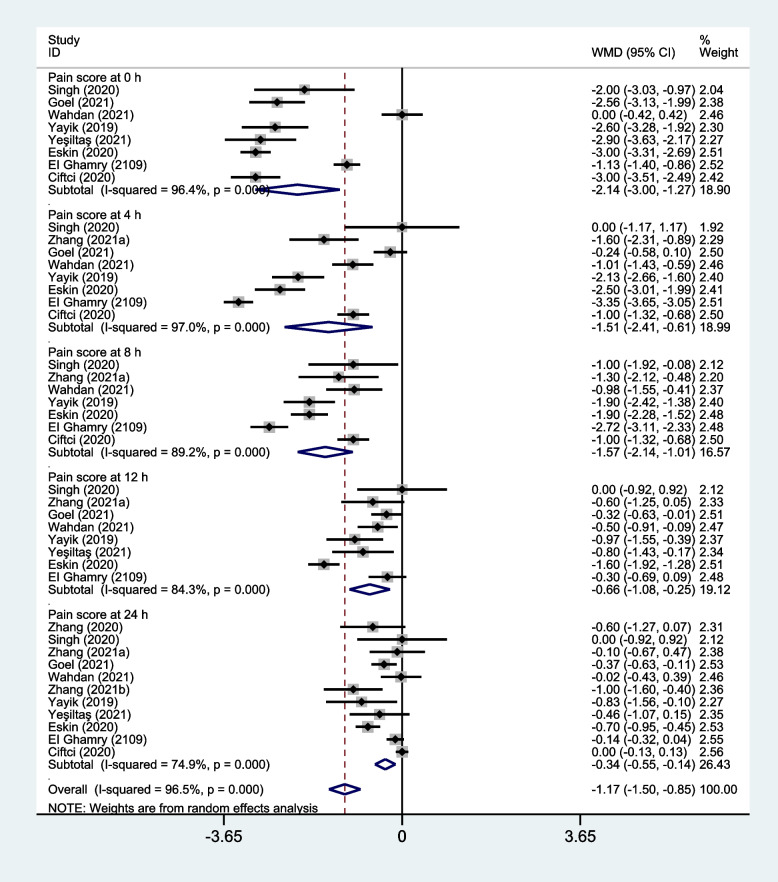
Forest plot of pain scores for the ESP block versus non-block care studies in the first 24 h after surgery. CI = confidence interval, WMD = weighted mean difference

Eleven studies were analyzed postoperative pain scores with the use of ESP block in patients receiving lumbar surgery [25–35]. Meta-analysis demonstrated that ESP block might significantly reduce pain scores at rest at various time points postoperatively compared with non-block group (Fig. 6): at 0 h (WMD, -2.14; 95% CI, -3.00 to-1.28; I^2^ = 96.4%; *P* < 0.001), at 4 h (WMD, -1.51; 95% CI, -2.41 to -0.61; I^2^ = 97.0%; *P* = 0.001), at 8 h (WMD, -1.57; 95% CI, -2.14 to -1.01; I^2^ = 89.2%; *P* < 0.001), at 12 h (WMD, -0.66; 95% CI, -1.08 to -0.25; I^2^ = 84.3%; *P* = 0.002) and at 24 h (WMD, -0.35; 95% CI, -0.55 to -0.14; I^2^ = 74.9%; *P* = 0.001). The ESP block might significantly reduce pain scores on movement at various time points postoperatively compared with non-block group (Fig. S2): at 0 h (WMD, -2.85; 95% CI, -3.27 to -2.43; I^2^ = 0.0%; *P* < 0.001), at 4 h (WMD, -1.59; 95% CI, -2.49 to -0.69; I^2^ = 87.7%; *P* = 0.001), at 8 h (WMD, -1.56; 95% CI, -2.33 to -0.79; I^2^ = 92.0%; *P* < 0.001), at 12 h (WMD, -0.96; 95% CI, -1.50 to -0.422; I^2^ = 79.8%; *P* < 0.001) and at 24 h (WMD, -0.65; 95% CI, -1.046 to -0.26; I^2^ = 72.3%; *P* = 0.001).

## First request for analgesia

**Fig. 7:**
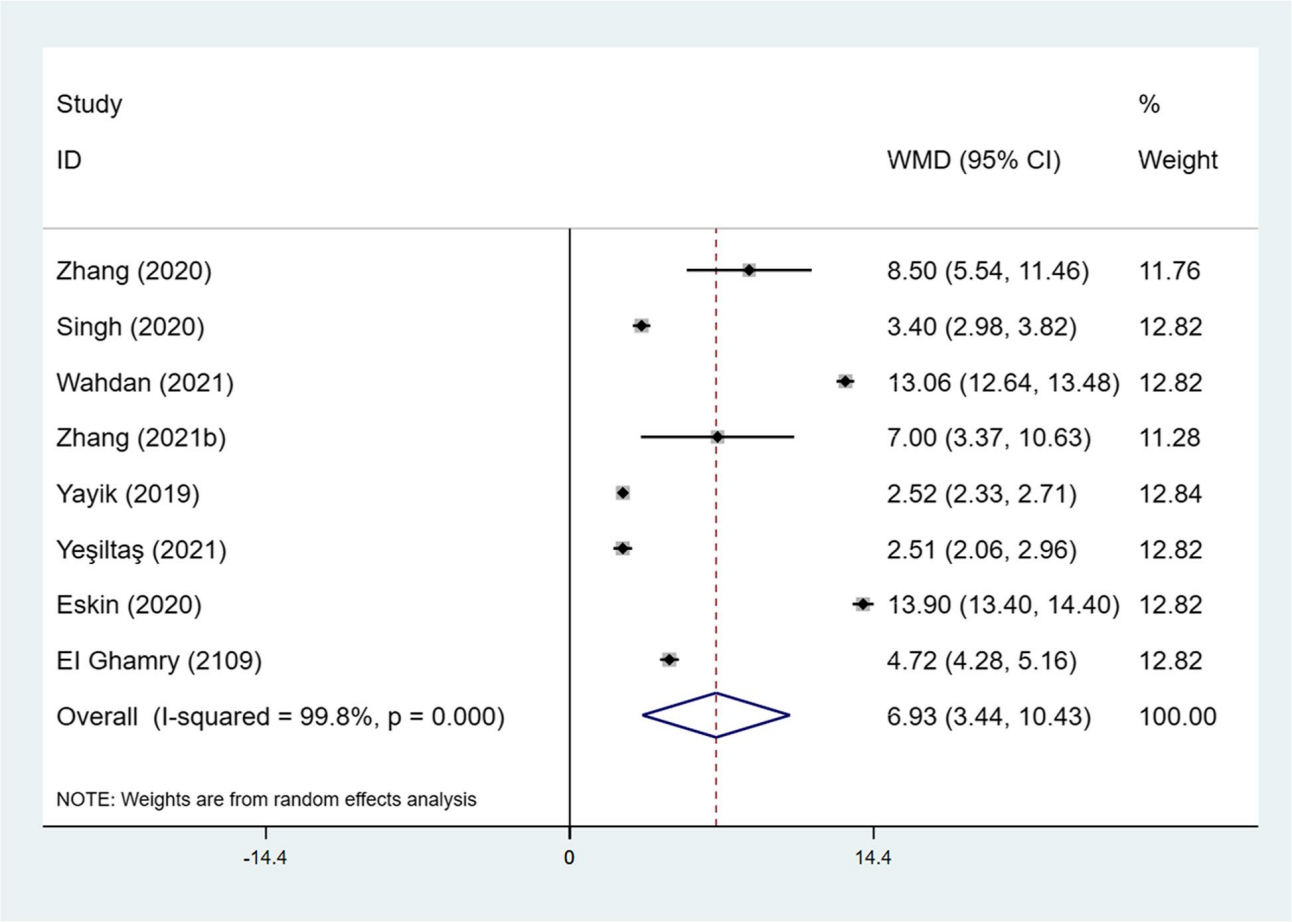
Forest plot of time to first request for analgesia. CI = confidence interval, WMD = weighted mean difference

First requests for analgesia were available in 8 studies [25, 26, 29–34]. On average, ESP block might delay the time to first request for analgesia by 6.93 h (95% CI: 3.44 to 10.43; I^2^ = 99.8%; *P* < 0.001) (Fig. 7). No evidence of publication bias was observed on Begg’s test (*P* = 0.39) or Egger test (*P* = 0.34) (Fig. S3). Sensitivity analysis and subgroup analysis did not significantly alter the summarized results (Fig. S4).

## Side effects and block-related complications

**Fig. 8:**
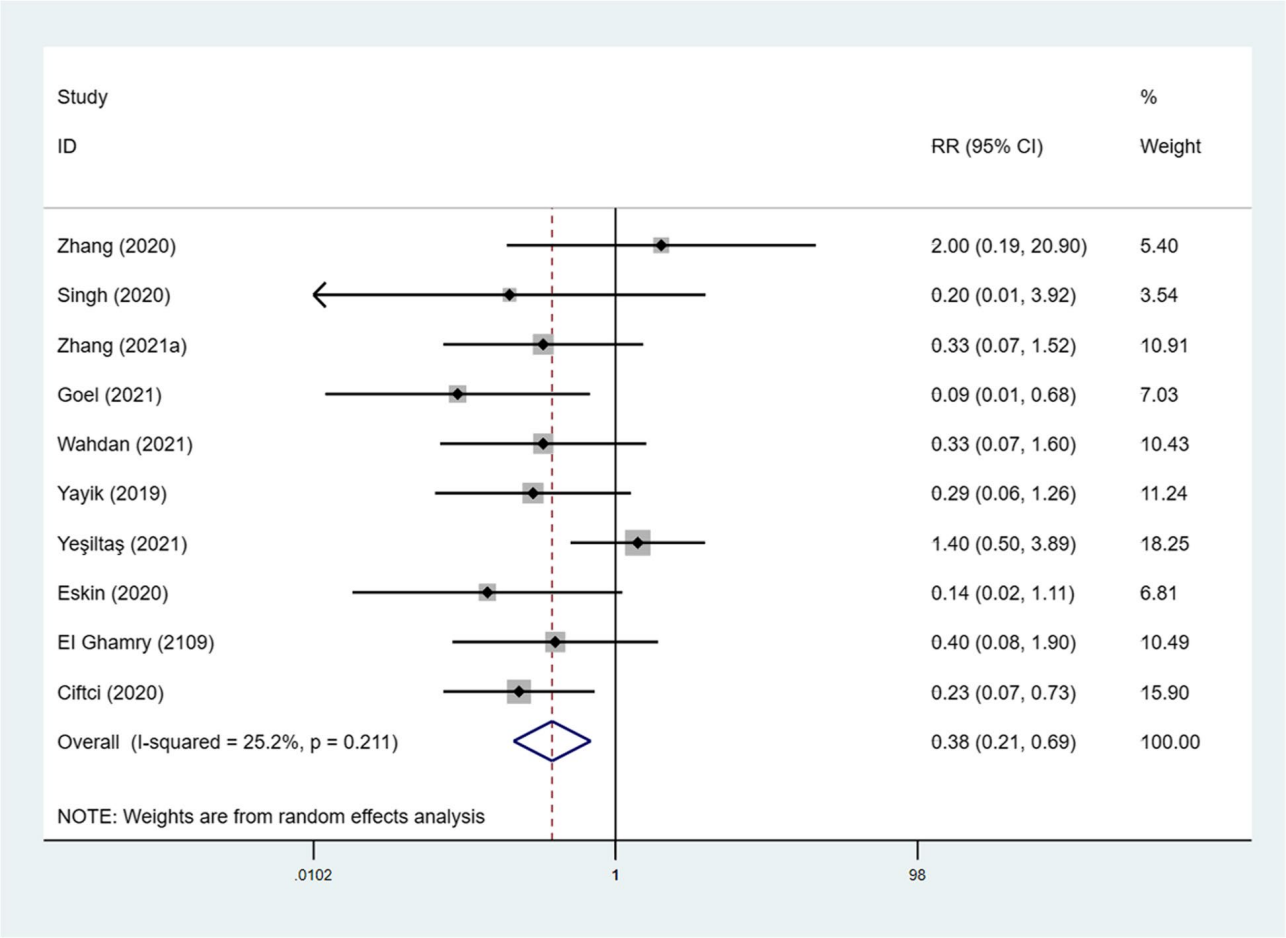
Forest plot for the comparison of the incidence of PONV. PONV = postoperative nausea and vomiting, RR = Risk ratio, CI = confidence interval

Ten studies included 716 patients and investigated the impact of the ESP block on the incidence of postoperative nausea and vomiting (PONV) in patients undergoing lumbar surgery [25–29, 31–35]. Notably, the ESP block significantly reduced the incidence of PONV (RR, 0.354; 95% CI, 0.23 to 0.56; I^2^ = 25.2%; *P* < 0.001) compared with non-block group (Fig. 8). Three studies assessed the incidence of pruritus [28, 33, 35], but there was no statistically significant difference between the 2 groups (Fig. S5). None of the patients in the reviewed trials experienced block-related complications.

## Discussion

The results of this meta-analysis demonstrate that the ESP block is more effective for postoperative analgesia in patients undergoing lumbar surgery. Performing ESP block led to a significant decrease in 24-h opioid consumption and was associated with less PONV. Furthermore, compared the non-block care, patients who received ESP block not only significantly reduced pain scores at postoperative time-points up to 24 h but also prolonged first analgesic requirement time by 6.93 h on average.

Lots of the patients experienced moderate-to-severe pain after lumbar spine surgery [36]. Finding a simple, efficient, and reliable postoperative analgesia with low side effects for major spinal surgeries remains a challenge for the anesthesiologist. Since 2016, the ESP block has been increasingly used range from the cervicothoracic to the lumbar spine region [6, 26, 31, 37–39]. The mechanism of action of lumbar ESP block remains unclear. A previous cadaveric study revealed that Lumbar ESP at L4 acted on the posterior branches of the spinal nerves, but seldom spread to the paravertebral space to block the spinal nerve [40]. In a recent cadaveric study, ESP block performed at L4, and the staining was found most cranially at L2 and extending caudally underneath the sacrum, to be confined to the posterior compartment of the spine sparing the nerve roots bilaterally [41]. Moreover, one reason for the popularity of the ESP block is that it is a simple sonographic identification of landmarks on ultrasound [25–27].

Several recent studies demonstrated that the use of ESP block leading to effective postoperative analgesia management in spinal surgeries [42–44]. Consistent with this finding, our study found that application of the ESP block in patients undergoing spinal surgery significantly reduced opioid consumption (morphine equivalent) by 8.7 mg in the first 24 h. In addition, our meta-analysis also revealed that ESP block was superior in reducing the incidence of PONV when compared to the control group. Notably, the opioid sparing effect was achieved with a significant reduction in PONV. In this meta-analysis, performing ESP block significantly decreased VAS pain scores at all time-points. More importantly, ESP block prolonged the time to first analgesic request by 6.93 h. Effective postoperative pain management leads to increased patient satisfaction [28, 29, 33].

This meta-analysis is subjected to several limitations worthy of consideration. First, high heterogeneity was found in some outcome measures. Although subgroup analysis (before or after induction) and sensitivity analysis were performed to identify the potential heterogeneity, we failed to change the heterogeneity. Second, the choice of local anesthetics (type, volume, and concentration) and different location (T12-L5) used for ESP block potentially contributed to the heterogeneity. However, it was not possible to perform a meta-regression to assess the impacts of these potential confounders due to the limited studies. Third, five included studies performed ESP block after general anesthesia induction [28, 29, 32, 33, 35]. The sensory perception of patients could not be assessed after block administration, which might contribute to the heterogeneity of the analysis. Fourth, because of ethical concerns about potential harm to patients, no sham injection was applied to the control group in nine included trials [25–28, 30, 31, 33–35]. This may have introduced bias. Fifth, different types of spinal surgery were included in this meta-analysis, and surgery types may be the source of this heterogeneity. However, subgroup and sensitivity analysis showed that there was no evidence for different surgery types in terms of opioid consumption and first analgesic request (Supplementary Table 4). Subsequently, further large sample multi-center studies are needed to prove those. Finally, no data were collected, we could not evaluate the effectiveness of ESP block in reducing chronic pain. Our protocol was not registered. These factors could affect our results.

In conclusion, our meta-analysis indicated that the ESP block significantly improved postoperative analgesia and reduced opioid consumption following spinal surgery compared with GA alone. Further studies are needed to investigate the safety and efficacy in these patients.

## Supplementary Information

**Additional file 1: Fig. S1.** Additional subgroup analysis of time of block.

**Additional file 2: Fig. S2.** Forest plot of pain scores on movement for the ESP block versus non-block care studies in the first 24 h after surgery.

**Additional file 3: Fig. S3.** Begg’s funnel plot of first request for analgesia.

**Additional file 4: Fig. S4.** Sensitivity analysis of first request for analgesia.

**Additional file 5: Fig. S5.** Forest plot for the comparison of the incidence of pruritus.

**Additional file 6: Supplementary table 1.** PRISMA checklist.

**Additional file 7: Supplementary Table 2.** Search strategies.

**Additional file 8: Supplementary Table 3.** Reasons for exclusion.

**Additional file 9: Supplementary Table 4.** Subgroup and sensitivity analysis for opioid consumption and first analgesic request.

## Data Availability

All data generated or analysed during this study are included in this published article [and its supplementary information files].

## Abbreviations

ESP: Erector spinae plane
RCTs: Randomized controlled trials
GA: General anesthesia
RRs: Risk ratios
WMDs: Weighted mean differences
PCIA: Patient-controlled intravenous analgesia
PONV: Postoperative nausea and vomiting
