# Supplementary figures and images for: Efficacy of erector spinae plane block for postoperative analgesia lumbar surgery: a systematic review and meta-analysis
Source: BMC Anesthesiol. 2023 Feb 16;23:54. doi: 10.1186/s12871-023-02013-3 (PMC9933390; doi:10.1186/s12871-023-02013-3)

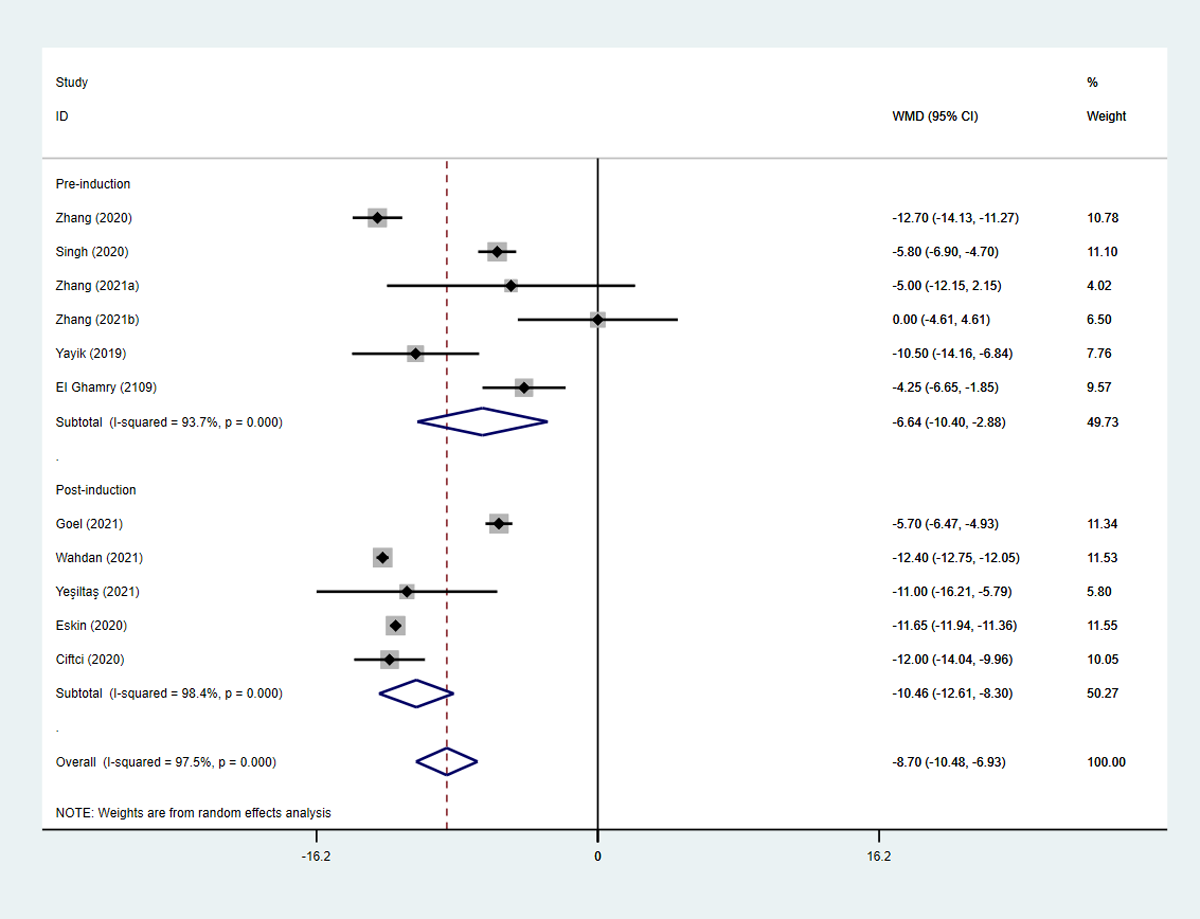

Supplement: Supplementary file 1 — Additional file 1: Fig. S1. Additional subgroup analysis of time of block. [file 12871_2023_2013_MOESM1_ESM.tif]

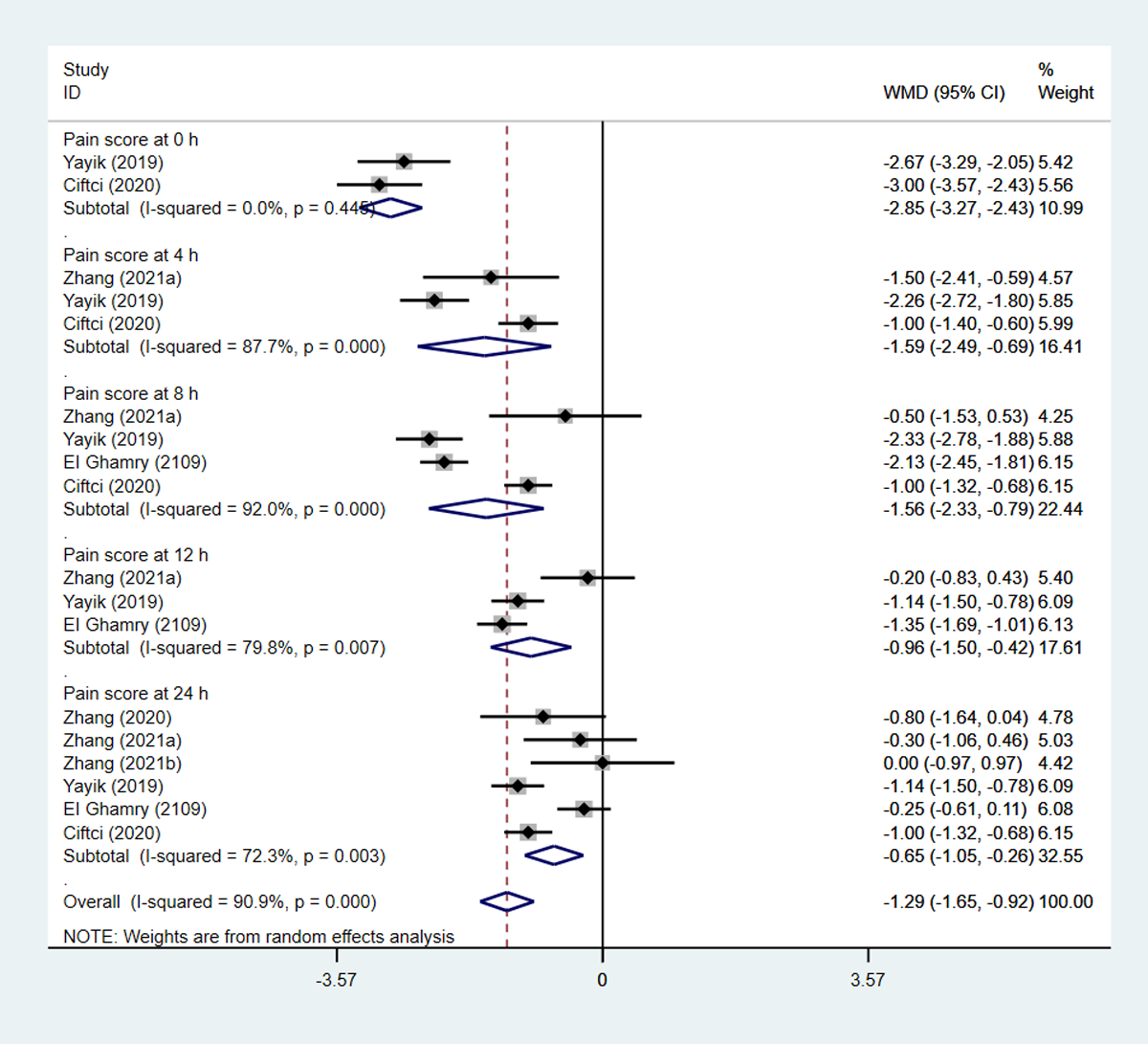

Supplement: Supplementary file 2 — Additional file 2: Fig. S2. Forest plot of pain scores on movement for the ESP block versus non-block care studies in the first 24 h after surgery. [file 12871_2023_2013_MOESM2_ESM.tif]

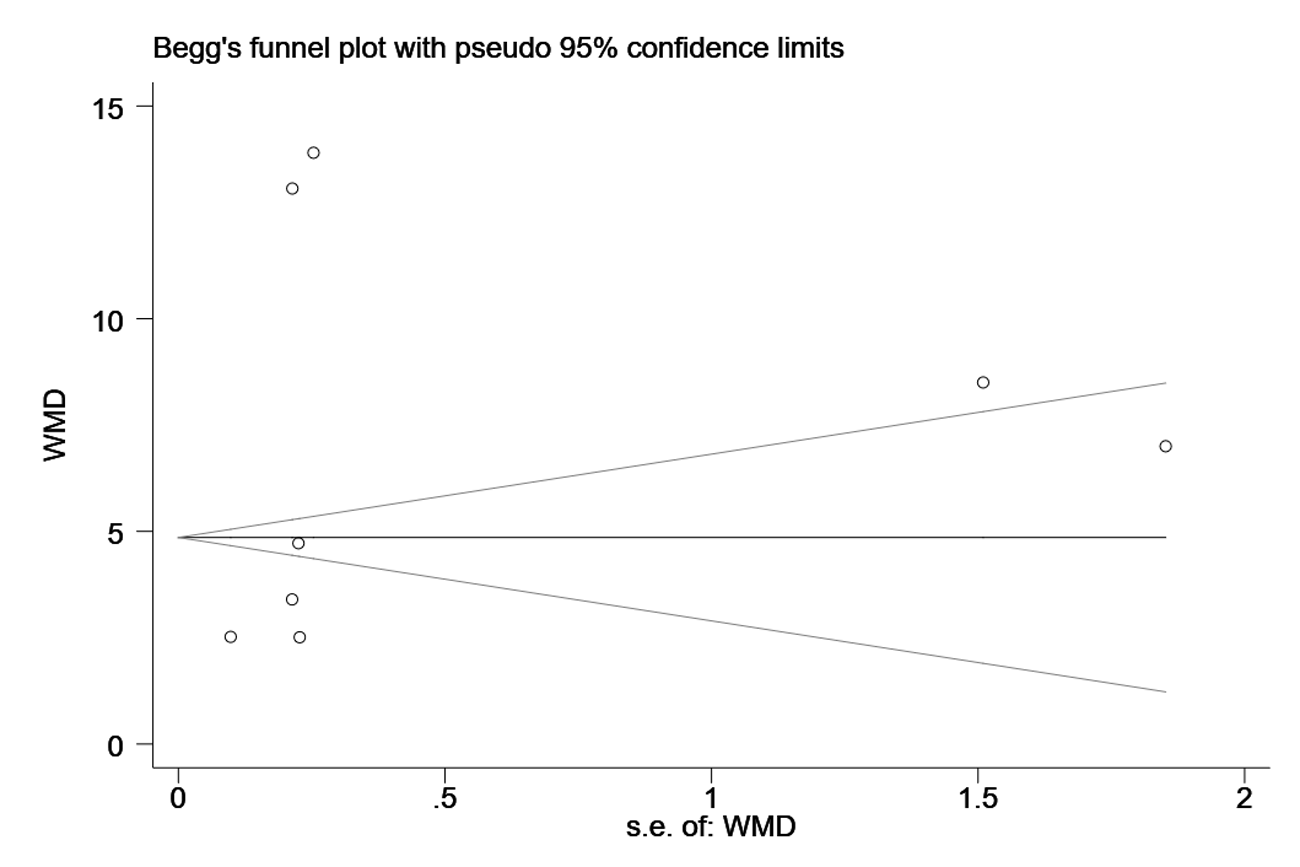

Supplement: Supplementary file 3 — Additional file 3: Fig. S3. Begg’s funnel plot of first request for analgesia. [file 12871_2023_2013_MOESM3_ESM.tif]

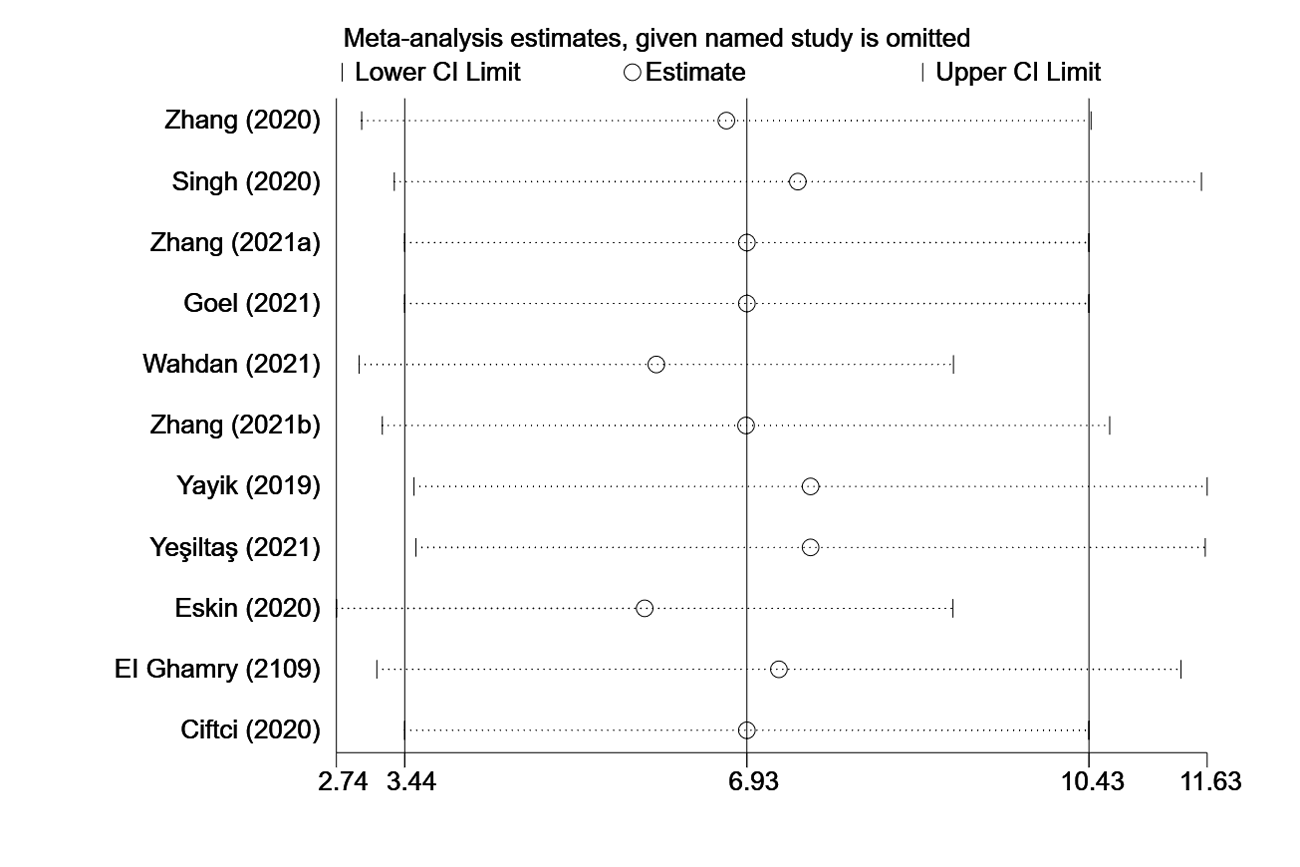

Supplement: Supplementary file 4 — Additional file 4: Fig. S4. Sensitivity analysis of first request for analgesia. [file 12871_2023_2013_MOESM4_ESM.tif]

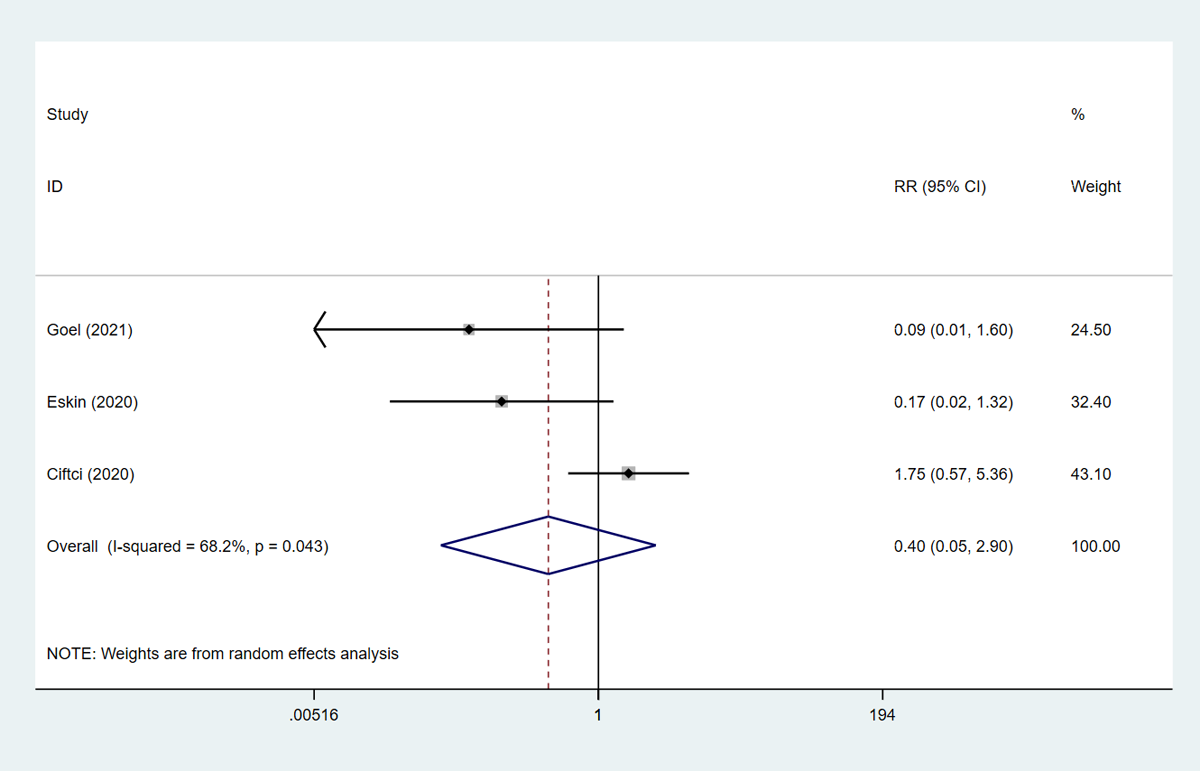

Supplement: Supplementary file 5 — Additional file 5: Fig. S5. Forest plot for the comparison of the incidence of pruritus. [file 12871_2023_2013_MOESM5_ESM.tif]
