# Supplementary material for: Efficacy of erector spinae plane block for postoperative analgesia lumbar surgery: a systematic review and meta-analysis
Source: BMC Anesthesiol. 2023 Feb 16;23:54. doi: 10.1186/s12871-023-02013-3 (PMC9933390; doi:10.1186/s12871-023-02013-3)
Supplement: Supplementary file 7 — Additional file 7: Supplementary Table 2. Search strategies. [file 12871_2023_2013_MOESM7_ESM.doc]

**Supplementary Table 2. Search strategies**

| **Search** | **Query** |
| --- | --- |
| #1 | Bilateral erector spinae plane block |
| #2 | Erector spinae plane block |
| #3 | Erector spinae plane nerve block |
| #4 | ESB block |
| #5 | ESBP |
| #6 | ESB |
| #7 | OR/#1-6 |
| #8 | Lumbar |
| #9 | Vertebral Column |
| #10 | Column, Vertebral |
| #11 | Columns, Vertebral |
| #12 | Vertebral Columns |
| #13 | Spinal Column |
| #14 | Column, Spinal |
| #15 | Columns, Spinal |
| #16 | Spinal Columns |
| #17 | Vertebra |
| #18 | Vertebrae |
| #19 | Lumbar Vertebrae |
| #20 | OR/#8-20 |
| #21 | Surgery |
| #22 | Operative therapy |
| #23 | Invasive procedures |
| #24 | Operative procedures |
| #25 | Operations |
| #26 | Perioperative procedures |
| #27 | Intraoperative procedures |
| #28 | Peroperative procedures |
| #29 | Preoperative procedures |
| #30 | OR/#21-29 |
| #31 | #7 AND #20AND#30 |
