# Supplementary material for: Efficacy of erector spinae plane block for postoperative analgesia lumbar surgery: a systematic review and meta-analysis
Source: BMC Anesthesiol. 2023 Feb 16;23:54. doi: 10.1186/s12871-023-02013-3 (PMC9933390; doi:10.1186/s12871-023-02013-3)
Supplement: Supplementary file 8 — Additional file 8: Supplementary Table 3. Reasons for exclusion. [file 12871_2023_2013_MOESM8_ESM.docx]

**Supplementary Table 3. Reasons for exclusion.**

| **Reasons for exclusion** | **Numbers** |
| --- | --- |
| Duplicate | 68 |
| Congress/ Conference abstract | 2 |
| Case report | 17 |
| Retrospective Study | 1 |
| Comparative study | 2 |
| Letter | 3 |
| Register of Controlled Trials | 8 |
| Human cadavers | 3 |
| Study protocol | 2 |
| Review | 4 |
