# Supplementary material for: Efficacy of erector spinae plane block for postoperative analgesia lumbar surgery: a systematic review and meta-analysis
Source: BMC Anesthesiol. 2023 Feb 16;23:54. doi: 10.1186/s12871-023-02013-3 (PMC9933390; doi:10.1186/s12871-023-02013-3)
Supplement: Supplementary file 9 — Additional file 9: Supplementary Table 4. Subgroup and sensitivity analysis for opioid consumption and first analgesic request. [file 12871_2023_2013_MOESM9_ESM.docx]

**Supplementary Table 4. subgroup and sensitivity analysis for opioid consumption and first analgesic request.**

| Subgroup | N | Effect size (95%CI) | P value | I^2^ |
| --- | --- | --- | --- | --- |
| **Opioid consumption** | 11 | -8.701(-10.476,—6.926) | <0.001 | 97.5% |
| Lumbar decompression surgery | 3 | -11.780(-12.387,-11.173) | <0.001 | 16.4% |
| Zhang (2020) omitted | 2 | -11.643(-11.936,-11.349) | <0.001 | 0.0% |
| Yayik (2019) omitted | 2 | -11.932(-12.845,-11.020) | <0.001 | 49.7% |
| Eskin (2020) omitted | 2 | -12.272(-13.979,-10.566) | <0.001 | 16.9% |
| Lumbar spine surgery | 3 | -6.466(-12.246,-0.686) | 0.028 | 98.7% |
| Singh (2020) omitted | 2 | -6.422(-18.566,5.722) | 0.300 | 96.4% |
| Wahdan (2021) omitted | 2 | -3.350(-8.965,2.265) | 0.242 | 82.6% |
| Zhang (2021b) omitted | 2 | -9.121(-15.589,-2.653) | 0.006 | 99.2% |
| Lumbar spinal fusion | 4 | -5.779(-7.632,-3.926) | <0.001 | 44.6% |
| Zhang (2021a) omitted | 3 | -5.937(-8.162,-3.713) | <0.001 | 62.8% |
| Goel (2021) omitted | 3 | -6.507(-10.943,-2.070) | 0.004 | 62.5% |
| Yeşiltaş (2021) omitted | 3 | -5.557(-6.289,-4.825) | <0.001 | 0.0% |
| EI Ghamry (2109) omitted | 3 | -6.880(-10.184,-3.577) | <0.001 | 49.3% |
| Lumbar discectomy | 1 | -12.000(-14.040,-9.960) | <0.001 | - |
| **First analgesic request** | 8 | 6.933(3.440,10.426) | <0.001 | 99.8% |
| Lumbar decompression surgery | 3 | 8.303(-0.747,17.353) | 0.072 | 99.9% |
| Zhang (2020) omitted | 2 | 8.208(-2.945,19.360) | 0.149 | 99.9% |
| Yayik (2019) omitted | 2 | 11.405(6.128,16.682) | <0.001 | 92.0% |
| Eskin (2020) omitted | 2 | 5.320(-0.528,11.169) | 0.075 | 93.6% |
| Lumbar spine surgery | 3 | 7.839(0.098,15.581) | 0.047 | 99.8% |
| Singh (2020) omitted | 2 | 10.309(4.396,16.223) | 0.001 | 90.5% |
| Wahdan (2021) omitted | 2 | 4.730(1.324,8.136) | 0.006 | 73.2% |
| Zhang (2021b) omitted | 2 | 8.230(-1.237,17.697) | 0.088 | 99.9% |
| Lumbar spinal fusion | 2 | 3.615(1.449,5.781) | 0.001 | 97.9% |
| Yeşiltaş (2021) omitted | 1 | 4.720(4.278,5.162) | - | - |
| EI Ghamry (2109) omitted | 1 | 2.510(2.064,2.956) | - | - |
